# *N*-(3-Methyl­phen­yl)benzene­sulfonamide

**DOI:** 10.1107/S1600536810002291

**Published:** 2010-01-23

**Authors:** B. Thimme Gowda, Sabine Foro, P. G. Nirmala, Hartmut Fuess

**Affiliations:** a Department of Chemistry, Mangalore University, Mangalagangotri 574 199, Mangalore, India; b Institute of Materials Science, Darmstadt University of Technology, Petersenstrasse 23, D-64287 Darmstadt, Germany

## Abstract

The asymmetric unit of the title compound, C_13_H_13_NO_2_S, contains two independent mol­ecules. The dihedral angles between the two aromatic rings are 67.9 (1) and 68.6 (1)° in the two mol­ecules. In the crystal, inter­molecular N—H⋯O hydrogen bonds link the mol­ecules into chains.

## Related literature

For the preparation of the title compound, see: Gowda *et al.* (2005 ▶). For related structures, see: Gelbrich *et al.* (2007 ▶; Gowda *et al.* (2008 ▶); Nirmala *et al.* (2009 ▶); Perlovich *et al.* (2006 ▶).
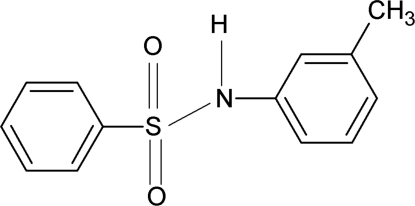


## Experimental

### 

#### Crystal data

C_13_H_13_NO_2_S
*M* *_r_* = 247.30
Orthorhombic, 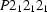

*a* = 8.787 (1) Å
*b* = 8.884 (1) Å
*c* = 32.406 (3) Å
*V* = 2529.7 (5) Å^3^
*Z* = 8
Cu *K*α radiation
μ = 2.19 mm^−1^
*T* = 299 K
0.60 × 0.60 × 0.35 mm

#### Data collection

Enraf–Nonius CAD-4 diffractometer
Absorption correction: ψ scan (North *et al.*, 1968 ▶) *T*_min_ = 0.353, *T*_max_ = 0.514
3284 measured reflections
3109 independent reflections
3002 reflections with *I* > 2σ(*I*)
*R*_int_ = 0.039
3 standard reflections every 120 min  intensity decay: 1.0%

#### Refinement

*R*[*F* ^2^ > 2σ(*F* ^2^)] = 0.033
*wR*(*F* ^2^) = 0.096
*S* = 1.01
3109 reflections
316 parameters
H atoms treated by a mixture of independent and constrained refinement
Δρ_max_ = 0.18 e Å^−3^
Δρ_min_ = −0.36 e Å^−3^
Absolute structure: Flack (1983 ▶), 507 Friedel pairs
Flack parameter: −0.010 (17)

### 

Data collection: *CAD-4-PC* (Enraf–Nonius, 1996 ▶); cell refinement: *CAD-4-PC*; data reduction: *REDU4* (Stoe & Cie, 1987 ▶); program(s) used to solve structure: *SHELXS97* (Sheldrick, 2008 ▶); program(s) used to refine structure: *SHELXL97* (Sheldrick, 2008 ▶); molecular graphics: *PLATON* (Spek, 2009 ▶); software used to prepare material for publication: *SHELXL97*.

## Supplementary Material

Crystal structure: contains datablocks I, global. DOI: 10.1107/S1600536810002291/bt5172sup1.cif

Structure factors: contains datablocks I. DOI: 10.1107/S1600536810002291/bt5172Isup2.hkl

Additional supplementary materials:  crystallographic information; 3D view; checkCIF report

## Figures and Tables

**Table 1:** Hydrogen-bond geometry (Å, °)

*D*—H⋯*A*	*D*—H	H⋯*A*	*D*⋯*A*	*D*—H⋯*A*
N1—H1*N*⋯O1^i^	0.87 (3)	2.08 (3)	2.919 (3)	162 (3)
N2—H2*N*⋯O3^ii^	0.82 (3)	2.17 (3)	2.981 (3)	178 (3)

Symmetry codes: (i) 
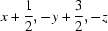
; (ii) 
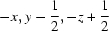
.

**Fig. 1.**
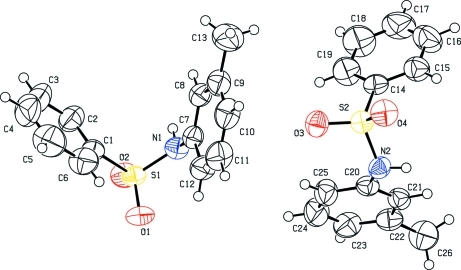
Molecular structure of the title compound, showing the atom labelling scheme and displacement ellipsoids drawn at the 50% probability level.

**Fig. 2.**
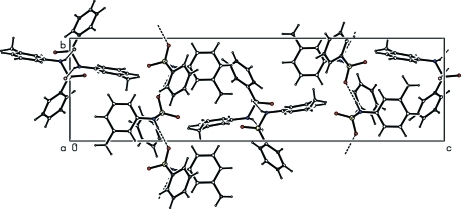
Molecular packing of the title compound with hydrogen bonding shown as dashed lines.

## supplementary crystallographic information

### Comment

In the present work, as a part of studying the effect of substituents on the crystal structures of *N*-(aryl)-arylsulfonamides (Gowda *et al.*, 2008; Nirmala *et al.*, 2009), the structure of *N*-(3-methylphenyl)benzenesulfonamide (I) has been determined. The asymmetric unit of (I) contains two independent molecules (Fig. 1). The conformations of the N—H bonds are *syn* to the *meta*- methyl groups in the aniline benzene rings, in contrast to the *anti* conformation observed with respect to the *ortho*-methyl group in *N*-(2-methylphenyl)benzenesulfonamide (II), to the *meta*-chloro group in *N*-(3-chlorophenyl)benzenesulfonamide(III)(Gowda *et al.*, 2008) and to the *meta*-methyl group in 4-methyl-*N*-(3-methylphenyl)benzenesulfonamide (IV) (Nirmala *et al.*, 2009).

The two benzene rings in (I) are tilted relative to each other by 67.9 (1)° in molecule 1 and 68.6 (1)° in molecule 2, compared to the values of 61.5 (1)° in (II), 65.4 (1)° in (III) and 83.9 (1)° in (IV),

The other bond parameters are similar to those observed in (II), (III), (IV) and other aryl sulfonamides (Perlovich *et al.*, 2006; Gelbrich *et al.*, 2007). The crystal packing stabilized by intermolecular N—H···O hydrogen bonds (Table 1) is shown in Fig. 2.

### Experimental

The solution of benzene (10 ml) in chloroform (40 ml) was treated dropwise with chlorosulfonic acid (25 ml) at 0 ° C. After the initial evolution of hydrogen chloride subsided, the reaction mixture was brought to room temperature and poured into crushed ice in a beaker. The chloroform layer was separated, washed with cold water and allowed to evaporate slowly. The residual benzenesulfonylchloride was treated with *m*-toluidine in the stoichiometric ratio and boiled for ten minutes. The reaction mixture was then cooled to room temperature and added to ice cold water (100 ml). The resultant solid *N*-(3-methylphenyl)benzenesulfonamide was filtered under suction and washed thoroughly with cold water. It was then recrystallized to constant melting point from dilute ethanol. The purity of the compound was checked and characterized by recording its infrared and NMR spectra (Gowda *et al.*, 2005).

The single crystals used in X-ray diffraction studies were grown in ethanolic solution by a slow evaporation at room temperature.

### Refinement

The H atoms of the NH groups were located in a difference map and their positional parameters were refined. The H-atoms bonded to C were positioned with idealized geometry using a riding model [C—H = 0.93–0.96 Å]. All H atoms were refined with isotropic displacement parameters  set to 1.2 times of the *U*_eq_ of the parent atom.

### Crystal data

**Table:**

C_13_H_13_NO_2_S	*F*(000) = 1040
*M**_r_* = 247.30	*D*_x_ = 1.299 Mg m^−^^3^
Orthorhombic, *P*2_1_2_1_2_1_	Cu *K*α radiation, λ = 1.54180 Å
Hall symbol: P 2ac 2ab	Cell parameters from 25 reflections
*a* = 8.787 (1) Å	θ = 6.5–20.2°
*b* = 8.884 (1) Å	µ = 2.19 mm^−^^1^
*c* = 32.406 (3) Å	*T* = 299 K
*V* = 2529.7 (5) Å^3^	Prism, colourless
*Z* = 8	0.60 × 0.60 × 0.35 mm

### Data collection

**Table:**

Enraf–Nonius CAD-4 diffractometer	3002 reflections with *I* > 2σ(*I*)
Radiation source: fine-focus sealed tube	*R*_int_ = 0.039
graphite	θ_max_ = 66.9°, θ_min_ = 2.7°
ω/2θ scans	*h* = −10→0
Absorption correction: ψ scan (North *et al.*, 1968)	*k* = −10→1
*T*_min_ = 0.353, *T*_max_ = 0.514	*l* = −38→4
3284 measured reflections	3 standard reflections every 120 min
3109 independent reflections	intensity decay: 1.0%

### Refinement

**Table:**

Refinement on *F*^2^	Hydrogen site location: inferred from neighbouring sites
Least-squares matrix: full	H atoms treated by a mixture of independent and constrained refinement
*R*[*F*^2^ > 2σ(*F*^2^)] = 0.033	*w* = 1/[σ^2^(*F*_o_^2^) + (0.0669*P*)^2^ + 0.4153*P*] where *P* = (*F*_o_^2^ + 2*F*_c_^2^)/3
*wR*(*F*^2^) = 0.096	(Δ/σ)_max_ < 0.001
*S* = 1.01	Δρ_max_ = 0.18 e Å^−^^3^
3109 reflections	Δρ_min_ = −0.36 e Å^−^^3^
316 parameters	Extinction correction: *SHELXL97* (Sheldrick, 2008), Fc^*^=kFc[1+0.001xFc^2^λ^3^/sin(2θ)]^-1/4^
0 restraints	Extinction coefficient: 0.0203 (7)
Primary atom site location: structure-invariant direct methods	Absolute structure: Flack (1983), 507 Friedel pairs
Secondary atom site location: difference Fourier map	Flack parameter: −0.010 (17)

### Special details

**Table:**

Geometry. All e.s.d.'s (except the e.s.d. in the dihedral angle between two l.s. planes) are estimated using the full covariance matrix. The cell e.s.d.'s are taken into account individually in the estimation of e.s.d.'s in distances, angles and torsion angles; correlations between e.s.d.'s in cell parameters are only used when they are defined by crystal symmetry. An approximate (isotropic) treatment of cell e.s.d.'s is used for estimating e.s.d.'s involving l.s. planes.
Refinement. Refinement of *F*^2^ against ALL reflections. The weighted *R*-factor *wR* and goodness of fit *S* are based on *F*^2^, conventional *R*-factors *R* are based on *F*, with *F* set to zero for negative *F*^2^. The threshold expression of *F*^2^ > σ(*F*^2^) is used only for calculating *R*-factors(gt) *etc*. and is not relevant to the choice of reflections for refinement. *R*-factors based on *F*^2^ are statistically about twice as large as those based on *F*, and *R*- factors based on ALL data will be even larger.

### Fractional atomic coordinates and isotropic or equivalent isotropic displacement parameters (Å^2^)

**Table:**

	*x*	*y*	*z*	*U*_iso_*/*U*_eq_	
S1	0.24673 (6)	0.87533 (7)	0.002545 (17)	0.05084 (19)	
O1	0.08754 (19)	0.8919 (2)	0.01175 (6)	0.0660 (5)	
O2	0.2926 (2)	0.8620 (3)	−0.03936 (5)	0.0675 (5)	
N1	0.3085 (2)	0.7245 (3)	0.02524 (6)	0.0534 (5)	
H1N	0.394 (4)	0.710 (4)	0.0126 (9)	0.064*	
C1	0.3398 (3)	1.0308 (3)	0.02455 (8)	0.0548 (6)	
C2	0.4797 (4)	1.0708 (4)	0.00878 (10)	0.0740 (8)	
H2	0.5241	1.0147	−0.0123	0.089*	
C3	0.5529 (6)	1.1951 (5)	0.02461 (13)	0.1007 (13)	
H3	0.6475	1.2232	0.0144	0.121*	
C4	0.4868 (7)	1.2767 (5)	0.05524 (16)	0.1167 (17)	
H4	0.5363	1.3615	0.0653	0.140*	
C5	0.3486 (6)	1.2364 (5)	0.07158 (15)	0.1124 (15)	
H5	0.3054	1.2924	0.0928	0.135*	
C6	0.2731 (4)	1.1093 (4)	0.05579 (10)	0.0816 (9)	
H6	0.1795	1.0794	0.0665	0.098*	
C7	0.3008 (3)	0.7054 (3)	0.06911 (7)	0.0502 (5)	
C8	0.4334 (3)	0.6721 (3)	0.09016 (8)	0.0563 (6)	
H8	0.5254	0.6688	0.0760	0.068*	
C9	0.4301 (4)	0.6434 (4)	0.13244 (8)	0.0675 (7)	
C10	0.2930 (4)	0.6565 (4)	0.15269 (9)	0.0758 (9)	
H10	0.2891	0.6403	0.1810	0.091*	
C11	0.1614 (4)	0.6932 (4)	0.13180 (10)	0.0793 (9)	
H11	0.0706	0.7034	0.1462	0.095*	
C12	0.1636 (3)	0.7147 (4)	0.08980 (9)	0.0671 (7)	
H12	0.0741	0.7352	0.0755	0.081*	
C13	0.5728 (5)	0.6023 (6)	0.15481 (11)	0.0973 (12)	
H13A	0.5927	0.4968	0.1513	0.117*	
H13B	0.5612	0.6242	0.1836	0.117*	
H13C	0.6564	0.6594	0.1439	0.117*	
S2	0.11402 (7)	0.27358 (7)	0.246445 (17)	0.05112 (19)	
O3	0.1367 (2)	0.4283 (2)	0.23607 (6)	0.0637 (5)	
O4	0.0977 (2)	0.2326 (2)	0.28860 (5)	0.0676 (5)	
N2	−0.0417 (2)	0.2159 (3)	0.22416 (6)	0.0527 (5)	
H2N	−0.065 (4)	0.136 (4)	0.2348 (8)	0.063*	
C14	0.2668 (3)	0.1716 (3)	0.22523 (7)	0.0532 (6)	
C15	0.2926 (3)	0.0270 (4)	0.23940 (9)	0.0674 (7)	
H15	0.2299	−0.0157	0.2593	0.081*	
C16	0.4143 (5)	−0.0526 (4)	0.22311 (12)	0.0902 (10)	
H16	0.4344	−0.1497	0.2323	0.108*	
C17	0.5046 (4)	0.0106 (5)	0.19371 (14)	0.0998 (13)	
H17	0.5863	−0.0438	0.1832	0.120*	
C18	0.4775 (5)	0.1509 (6)	0.17945 (14)	0.1068 (14)	
H18	0.5396	0.1913	0.1590	0.128*	
C19	0.3571 (4)	0.2356 (4)	0.19508 (10)	0.0821 (9)	
H19	0.3381	0.3324	0.1855	0.099*	
C20	−0.0626 (3)	0.2246 (3)	0.18014 (7)	0.0504 (5)	
C21	−0.1049 (3)	0.0949 (3)	0.15949 (8)	0.0539 (6)	
H21	−0.1118	0.0046	0.1739	0.065*	
C22	−0.1374 (3)	0.0981 (4)	0.11760 (8)	0.0609 (6)	
C23	−0.1195 (4)	0.2328 (4)	0.09707 (9)	0.0729 (8)	
H23	−0.1369	0.2369	0.0688	0.087*	
C24	−0.0769 (5)	0.3601 (4)	0.11743 (10)	0.0847 (10)	
H24	−0.0661	0.4496	0.1028	0.102*	
C25	−0.0492 (4)	0.3584 (4)	0.15961 (9)	0.0705 (8)	
H25	−0.0223	0.4460	0.1735	0.085*	
C26	−0.1914 (5)	−0.0405 (4)	0.09576 (11)	0.0861 (10)	
H26A	−0.1587	−0.1280	0.1107	0.103*	
H26B	−0.1496	−0.0429	0.0684	0.103*	
H26C	−0.3005	−0.0394	0.0942	0.103*	

### Atomic displacement parameters (Å^2^)

**Table:**

	*U*^11^	*U*^22^	*U*^33^	*U*^12^	*U*^13^	*U*^23^
S1	0.0439 (3)	0.0637 (3)	0.0449 (3)	−0.0013 (3)	−0.0050 (2)	−0.0031 (3)
O1	0.0395 (8)	0.0884 (13)	0.0702 (11)	0.0026 (9)	−0.0048 (8)	−0.0042 (11)
O2	0.0692 (12)	0.0883 (13)	0.0450 (8)	−0.0003 (11)	−0.0035 (8)	−0.0041 (10)
N1	0.0497 (10)	0.0613 (12)	0.0492 (10)	0.0043 (10)	0.0060 (9)	−0.0033 (10)
C1	0.0554 (14)	0.0568 (13)	0.0523 (13)	−0.0011 (12)	−0.0138 (11)	0.0063 (12)
C2	0.0700 (17)	0.0829 (19)	0.0690 (18)	−0.0202 (17)	−0.0053 (14)	0.0104 (16)
C3	0.107 (3)	0.095 (3)	0.101 (3)	−0.044 (2)	−0.021 (2)	0.017 (2)
C4	0.139 (4)	0.073 (2)	0.138 (4)	−0.029 (3)	−0.054 (3)	0.003 (3)
C5	0.130 (4)	0.090 (3)	0.117 (3)	0.014 (3)	−0.031 (3)	−0.038 (3)
C6	0.083 (2)	0.082 (2)	0.0793 (18)	0.0032 (19)	−0.0102 (17)	−0.0229 (18)
C7	0.0525 (12)	0.0477 (12)	0.0503 (11)	−0.0035 (11)	0.0060 (10)	−0.0011 (11)
C8	0.0538 (14)	0.0587 (14)	0.0564 (13)	−0.0038 (12)	0.0040 (11)	0.0002 (12)
C9	0.0789 (18)	0.0656 (17)	0.0580 (14)	−0.0086 (16)	−0.0088 (14)	0.0048 (14)
C10	0.097 (2)	0.0811 (19)	0.0496 (13)	−0.0142 (19)	0.0120 (15)	0.0081 (15)
C11	0.078 (2)	0.091 (2)	0.0682 (17)	−0.0070 (19)	0.0266 (16)	0.0095 (17)
C12	0.0560 (14)	0.0802 (18)	0.0651 (15)	−0.0021 (15)	0.0115 (13)	0.0086 (15)
C13	0.093 (3)	0.120 (3)	0.079 (2)	0.001 (3)	−0.0190 (19)	0.022 (2)
S2	0.0540 (3)	0.0561 (3)	0.0432 (3)	0.0010 (3)	0.0032 (2)	−0.0055 (2)
O3	0.0722 (12)	0.0541 (9)	0.0649 (11)	−0.0015 (9)	0.0044 (9)	−0.0077 (8)
O4	0.0777 (12)	0.0808 (12)	0.0444 (8)	0.0005 (12)	0.0036 (8)	−0.0037 (9)
N2	0.0503 (10)	0.0584 (11)	0.0493 (10)	−0.0057 (10)	0.0036 (9)	0.0040 (10)
C14	0.0456 (12)	0.0639 (14)	0.0500 (12)	0.0015 (11)	−0.0032 (11)	−0.0098 (11)
C15	0.0641 (17)	0.0689 (16)	0.0693 (16)	0.0066 (14)	0.0029 (13)	−0.0031 (15)
C16	0.086 (2)	0.077 (2)	0.107 (3)	0.025 (2)	−0.005 (2)	−0.007 (2)
C17	0.068 (2)	0.106 (3)	0.125 (3)	0.024 (2)	0.017 (2)	−0.020 (3)
C18	0.082 (2)	0.119 (3)	0.119 (3)	0.020 (3)	0.045 (2)	0.011 (3)
C19	0.0743 (19)	0.085 (2)	0.087 (2)	0.0097 (19)	0.0281 (17)	0.0082 (19)
C20	0.0423 (11)	0.0606 (14)	0.0483 (11)	0.0029 (11)	0.0021 (9)	0.0032 (12)
C21	0.0503 (12)	0.0555 (13)	0.0561 (12)	0.0054 (12)	0.0044 (11)	0.0010 (11)
C22	0.0504 (13)	0.0743 (16)	0.0579 (13)	0.0047 (13)	0.0017 (11)	−0.0086 (13)
C23	0.0739 (18)	0.093 (2)	0.0519 (13)	−0.009 (2)	−0.0078 (13)	0.0054 (15)
C24	0.100 (3)	0.087 (2)	0.0675 (17)	−0.018 (2)	−0.0173 (18)	0.0265 (17)
C25	0.085 (2)	0.0646 (16)	0.0625 (15)	−0.0112 (16)	−0.0115 (15)	0.0075 (14)
C26	0.097 (2)	0.082 (2)	0.0790 (19)	0.002 (2)	−0.0076 (19)	−0.0239 (18)

### Geometric parameters (Å, °)

**Table:**

S1—O2	1.4214 (18)	S2—O4	1.4212 (18)
S1—O1	1.4378 (18)	S2—O3	1.429 (2)
S1—N1	1.622 (2)	S2—N2	1.630 (2)
S1—C1	1.756 (3)	S2—C14	1.760 (3)
N1—C7	1.433 (3)	N2—C20	1.440 (3)
N1—H1N	0.87 (3)	N2—H2N	0.82 (3)
C1—C6	1.362 (4)	C14—C19	1.381 (4)
C1—C2	1.379 (4)	C14—C15	1.383 (4)
C2—C3	1.377 (5)	C15—C16	1.386 (5)
C2—H2	0.9300	C15—H15	0.9300
C3—C4	1.359 (6)	C16—C17	1.361 (6)
C3—H3	0.9300	C16—H16	0.9300
C4—C5	1.373 (7)	C17—C18	1.350 (6)
C4—H4	0.9300	C17—H17	0.9300
C5—C6	1.406 (6)	C18—C19	1.393 (5)
C5—H5	0.9300	C18—H18	0.9300
C6—H6	0.9300	C19—H19	0.9300
C7—C12	1.382 (4)	C20—C25	1.367 (4)
C7—C8	1.382 (4)	C20—C21	1.383 (4)
C8—C9	1.394 (3)	C21—C22	1.387 (3)
C8—H8	0.9300	C21—H21	0.9300
C9—C10	1.377 (5)	C22—C23	1.378 (4)
C9—C13	1.494 (5)	C22—C26	1.497 (4)
C10—C11	1.379 (5)	C23—C24	1.362 (5)
C10—H10	0.9300	C23—H23	0.9300
C11—C12	1.375 (4)	C24—C25	1.388 (4)
C11—H11	0.9300	C24—H24	0.9300
C12—H12	0.9300	C25—H25	0.9300
C13—H13A	0.9600	C26—H26A	0.9600
C13—H13B	0.9600	C26—H26B	0.9600
C13—H13C	0.9600	C26—H26C	0.9600
			
O2—S1—O1	118.86 (11)	O4—S2—O3	119.12 (12)
O2—S1—N1	105.62 (12)	O4—S2—N2	105.12 (12)
O1—S1—N1	108.43 (12)	O3—S2—N2	108.38 (12)
O2—S1—C1	108.75 (13)	O4—S2—C14	108.70 (12)
O1—S1—C1	106.75 (13)	O3—S2—C14	107.27 (12)
N1—S1—C1	108.04 (11)	N2—S2—C14	107.80 (12)
C7—N1—S1	122.14 (18)	C20—N2—S2	121.91 (17)
C7—N1—H1N	119.3 (19)	C20—N2—H2N	116 (2)
S1—N1—H1N	102 (2)	S2—N2—H2N	107 (2)
C6—C1—C2	121.8 (3)	C19—C14—C15	121.6 (3)
C6—C1—S1	120.3 (2)	C19—C14—S2	120.2 (2)
C2—C1—S1	117.9 (2)	C15—C14—S2	118.3 (2)
C3—C2—C1	119.0 (4)	C14—C15—C16	118.3 (3)
C3—C2—H2	120.5	C14—C15—H15	120.9
C1—C2—H2	120.5	C16—C15—H15	120.9
C4—C3—C2	120.0 (4)	C17—C16—C15	120.4 (4)
C4—C3—H3	120.0	C17—C16—H16	119.8
C2—C3—H3	120.0	C15—C16—H16	119.8
C3—C4—C5	121.4 (4)	C18—C17—C16	121.1 (4)
C3—C4—H4	119.3	C18—C17—H17	119.4
C5—C4—H4	119.3	C16—C17—H17	119.4
C4—C5—C6	119.1 (4)	C17—C18—C19	120.5 (4)
C4—C5—H5	120.4	C17—C18—H18	119.7
C6—C5—H5	120.4	C19—C18—H18	119.7
C1—C6—C5	118.6 (4)	C14—C19—C18	118.1 (4)
C1—C6—H6	120.7	C14—C19—H19	121.0
C5—C6—H6	120.7	C18—C19—H19	121.0
C12—C7—C8	120.6 (2)	C25—C20—C21	120.8 (2)
C12—C7—N1	121.0 (2)	C25—C20—N2	121.2 (3)
C8—C7—N1	118.4 (2)	C21—C20—N2	117.9 (2)
C7—C8—C9	120.5 (3)	C20—C21—C22	120.8 (2)
C7—C8—H8	119.8	C20—C21—H21	119.6
C9—C8—H8	119.8	C22—C21—H21	119.6
C10—C9—C8	118.1 (3)	C23—C22—C21	117.8 (3)
C10—C9—C13	121.6 (3)	C23—C22—C26	121.5 (3)
C8—C9—C13	120.3 (3)	C21—C22—C26	120.7 (3)
C9—C10—C11	121.3 (2)	C24—C23—C22	121.2 (3)
C9—C10—H10	119.4	C24—C23—H23	119.4
C11—C10—H10	119.4	C22—C23—H23	119.4
C12—C11—C10	120.5 (3)	C23—C24—C25	121.1 (3)
C12—C11—H11	119.7	C23—C24—H24	119.5
C10—C11—H11	119.7	C25—C24—H24	119.5
C11—C12—C7	118.9 (3)	C20—C25—C24	118.3 (3)
C11—C12—H12	120.5	C20—C25—H25	120.9
C7—C12—H12	120.5	C24—C25—H25	120.9
C9—C13—H13A	109.5	C22—C26—H26A	109.5
C9—C13—H13B	109.5	C22—C26—H26B	109.5
H13A—C13—H13B	109.5	H26A—C26—H26B	109.5
C9—C13—H13C	109.5	C22—C26—H26C	109.5
H13A—C13—H13C	109.5	H26A—C26—H26C	109.5
H13B—C13—H13C	109.5	H26B—C26—H26C	109.5
			
O2—S1—N1—C7	172.1 (2)	O4—S2—N2—C20	−174.2 (2)
O1—S1—N1—C7	−59.5 (2)	O3—S2—N2—C20	57.4 (2)
C1—S1—N1—C7	55.8 (2)	C14—S2—N2—C20	−58.4 (3)
O2—S1—C1—C6	150.7 (2)	O4—S2—C14—C19	−146.4 (3)
O1—S1—C1—C6	21.3 (3)	O3—S2—C14—C19	−16.4 (3)
N1—S1—C1—C6	−95.2 (3)	N2—S2—C14—C19	100.1 (3)
O2—S1—C1—C2	−28.4 (3)	O4—S2—C14—C15	33.9 (2)
O1—S1—C1—C2	−157.8 (2)	O3—S2—C14—C15	163.9 (2)
N1—S1—C1—C2	85.8 (2)	N2—S2—C14—C15	−79.6 (2)
C6—C1—C2—C3	−1.1 (5)	C19—C14—C15—C16	1.2 (4)
S1—C1—C2—C3	178.0 (3)	S2—C14—C15—C16	−179.1 (3)
C1—C2—C3—C4	−0.3 (5)	C14—C15—C16—C17	−0.5 (5)
C2—C3—C4—C5	1.3 (7)	C15—C16—C17—C18	−0.6 (7)
C3—C4—C5—C6	−1.0 (7)	C16—C17—C18—C19	1.0 (8)
C2—C1—C6—C5	1.4 (5)	C15—C14—C19—C18	−0.8 (5)
S1—C1—C6—C5	−177.7 (3)	S2—C14—C19—C18	179.5 (3)
C4—C5—C6—C1	−0.3 (6)	C17—C18—C19—C14	−0.3 (7)
S1—N1—C7—C12	57.0 (3)	S2—N2—C20—C25	−56.3 (3)
S1—N1—C7—C8	−125.3 (2)	S2—N2—C20—C21	127.3 (2)
C12—C7—C8—C9	1.7 (4)	C25—C20—C21—C22	−1.1 (4)
N1—C7—C8—C9	−176.1 (3)	N2—C20—C21—C22	175.4 (2)
C7—C8—C9—C10	−3.1 (5)	C20—C21—C22—C23	2.8 (4)
C7—C8—C9—C13	177.9 (3)	C20—C21—C22—C26	−176.7 (3)
C8—C9—C10—C11	1.6 (5)	C21—C22—C23—C24	−2.4 (5)
C13—C9—C10—C11	−179.4 (4)	C26—C22—C23—C24	177.1 (3)
C9—C10—C11—C12	1.3 (6)	C22—C23—C24—C25	0.3 (6)
C10—C11—C12—C7	−2.8 (5)	C21—C20—C25—C24	−1.0 (5)
C8—C7—C12—C11	1.2 (5)	N2—C20—C25—C24	−177.4 (3)
N1—C7—C12—C11	178.9 (3)	C23—C24—C25—C20	1.4 (5)

### Hydrogen-bond geometry (Å, °)

**Table:**

*D*—H···*A*	*D*—H	H···*A*	*D*···*A*	*D*—H···*A*
N1—H1N···O1^i^	0.87 (3)	2.08 (3)	2.919 (3)	162 (3)
N2—H2N···O3^ii^	0.82 (3)	2.17 (3)	2.981 (3)	178 (3)

Symmetry codes: (i) *x*+1/2, −*y*+3/2, −*z*; (ii) −*x*, *y*−1/2, −*z*+1/2.
